# Modified Polymeric Biosorbents from *Rumex acetosella* for the Removal of Heavy Metals in Wastewater

**DOI:** 10.3390/polym14112191

**Published:** 2022-05-28

**Authors:** Carlos A. Ligarda-Samanez, David Choque-Quispe, Henry Palomino-Rincón, Betsy S. Ramos-Pacheco, Elibet Moscoso-Moscoso, Mary L. Huamán-Carrión, Diego E. Peralta-Guevara, Mirian E. Obregón-Yupanqui, Jimmy Aroni-Huamán, Eyner Y. Bravo-Franco, Wilbert Palomino-Rincón, Germán De la Cruz

**Affiliations:** 1 Food Nanotechnology Research Laboratory, Universidad Nacional José María Arguedas, Andahuaylas 03701, Peru; elibetmm22@gmail.com (E.M.-M.); mhuaman@unajma.edu.pe (M.L.H.-C.); 2 Water Analysis and Control Research Laboratory, Universidad Nacional José María Arguedas, Andahuaylas 03701, Peru; dchoque@unajma.edu.pe (D.C.-Q.); diepltagvra@gmail.com (D.E.P.-G.); 3 Agroindustrial Engineering, Universidad Nacional José María Arguedas, Andahuaylas 03701, Peru; hpalomino@unajma.edu.pe (H.P.-R.); bsramos@unajma.edu.pe (B.S.R.-P.); mirianobregon95@gmail.com (M.E.O.-Y.); jaroni@unajma.edu.pe (J.A.-H.); 4 Faculty of Business Sciences, Universidad Nacional José María Arguedas, Andahuaylas 03701, Peru; ebravo@unajma.edu.pe; 5 Agricultural and Livestock Engineering, Universidad Nacional San Antonio Abad, Cusco 08000, Peru; wilbert.palomino@unsaac.edu.pe; 6 Agricultural Science Facultad, Universidad Nacional San Cristobal de Huamanga, Ayacucho 05000, Peru; german.delacruz@unsch.edu.pe

**Keywords:** biosorption, heavy metals, modified biomass, metal removal, *Rumex acetosella*

## Abstract

The contamination of water resources by effluents from various industries often contains heavy metals, which cause irreversible damage to the environment and health. The objective was to evaluate different biosorbents from the weed *Rumex acetosella* to remove metal cations in wastewater. Drying, grinding and sieving of the stems was carried out to obtain the biomass, retaining the fractions of 250 to 500 µm and 500 to 750 µm, which served to obtain the biosorbents in natura (unmodified), acidic, alkaline, and mixed. Proximal analysis, PZC, TOC, removal capacity, influence of pH, functional groups, thermal analysis, structural characteristics, adsorption isotherms, and kinetic study were evaluated. The 250 µm mixed treatment was the one that presented the highest removal percentages, mainly due to the OH, NH, -C-H, COOH, and C-O functional groups achieving the removal of up to 96.14% of lead, 36.30% of zinc, 34.10% of cadmium and 32.50% of arsenic. For contact times of 120 min and an optimum pH of 5.0, a loss of cellulose mass of 59% at 328 °C and a change in the surface of the material were also observed, which allowed for obtaining a topography with greater chelating capacity, and the Langmuir and pseudo-second order models were better fitted to the adsorption data. The new biosorbents could be used in wastewater treatment economically and efficiently.

## 1. Introduction

The different economic activities that man develops for his subsistence usually generate wastewater; in many cases, these effluents contain heavy metals in their composition, which cause an adverse effect on the environment and human health. Currently, the world population requires greater access to clean and safe water, and this need will increase rapidly in the coming years. It is in this context, that research on various raw materials is necessary to obtain novel biosorbents that allow for the removal of heavy metals in a more versatile and less expensive way [1,2,3].

Some of the main industries that produce this type of pollutant are mining, metallurgical and tannery [4,5], whose waste must be treated properly due to its high content of pollutants and the irreversible damage it causes to ecosystems [6]. The metallic cations that are most frequently found in contaminated effluents are arsenic (As), which comes from mining activities, metal smelters, and fossil combustion; it has carcinogenic effects on the skin, heart, kidneys, and gastrointestinal tract [7,8]; cadmium (Cd), whose main source is metal processing, battery recycling and fossil fuel burning industries, causes damage to the bone, cardiovascular and renal system of the human being [8,9]; lead (Pb), which results from mining, steel, automotive, battery and paint processing, has harmful effects on the central nervous system and kidneys, causes liver damage and causes memory problems, in addition, it causes problems with growth, hearing and anemia, also generating problems of arterial hypertension and cardiovascular damage in adults [8,10]; zinc (Zn), which comes from the pharmaceutical industry, as well as the production of galvanized products, dyes, pesticides and cosmetics, causes abdominal pain, anemia in children and phytotoxicity [8,11,12].

Among the most economical methods for the treatment of water contaminated with heavy metals is biosorption; this technique has advantages in its applicability, easy obtaining, simple design, and wide use [13,14,15,16,17,18]; the physicochemical characteristics of biosorbents rich in lignocellulose allow us to understand the different adsorption mechanisms and their potential application in the removal of metal cations; Characterizations such as proximal, thermal, chemical groups, surface, geometry, and porosity analysis are important for this purpose. It is also known that lignocellulose is present in different plant fibers and also has functional groups such as hydroxyl, carboxyl, and silanol, which contribute to its high biosorption capacity [19,20]. On the other hand, it could be possible to treat wastewater using other techniques and polymeric matrices, such as spent coffee [21], plastic shavings and loofah [22], non-living cells of *Nannochloropsis oceanica* [23], agro-industrial wastes [24] and cacti [25].

The use of various biomaterials as absorbents allows for the selective removal of metal ions in wastewater [26,27,28]. However, no information has been reported on the use of a weed such as *Rumex acetosella* to obtain biosorbent material and its potential use in the treatment of contaminated water. It is known that *R. acetosella* easily invades crop fields that are free due to its great colonization capacity, and it is also widely distributed throughout the world [29]. This plant is considered a problem and causes economic losses in agriculture since it interferes with agricultural work, growing in places where it is not wanted. Taking into account the above, modified biosorbents could be prepared from this material, which has no commercial value, in a simple and cost-effective way [8,30].

**Figure 1:**
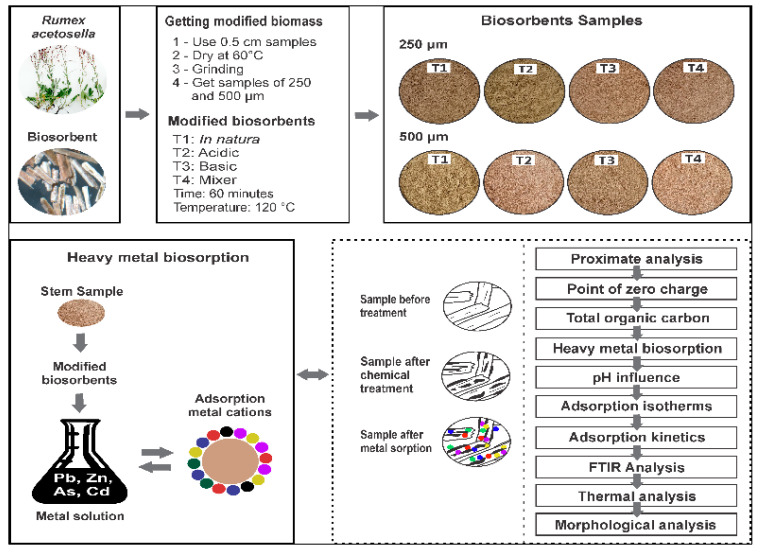
Experimental flow diagram.

In the present investigation, it is proposed to obtain and characterize new biosorbents using a source rich in fiber, such as the stem of *R. acetosella*, carrying out this purpose chemical modification at a low cost, which allows for high rates of removal of heavy metals in a profitable way and eco-sustainability in different residual effluents [31]. The complete experimental flow chart is shown in Figure 1.

## 2. Materials and Methods

### 2.1. Vegetal Material

Samples of *R. acetosella* were collected in cultivated fields located at 3904 m altitude, whose coordinates were 13°40′15″ S and 73°14′24″ W, in the community of Chulcuisa, district of San Jerónimo, Province of Andahuaylas, Peru. The plant material was collected in January and February 2021 and kindly provided by local farmers; the collected samples were stored in plastic containers at room temperature for later use in the experiments.

### 2.2. Proximate Analysis of Vegetal Material 

It was determined in dry samples according to the standard methods of the Association of Official Analytical Chemists (AOAC) [32] for moisture (AOAC 925.10), protein (AOAC 2003.05), fat (AOAC, 923.03), ash (AOAC 960.52) and fiber (AOAC 962.09). Carbohydrates were determined by difference.

### 2.3. Getting Modified Biomass 

**Figure 2:**
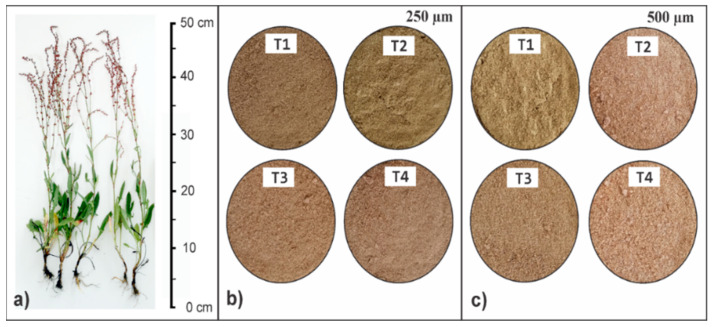
Plant material (**a**) and modified biosorbents from *R. acetosella* 250 µm (**b**) and 500 µm (**c**).

The *R. acetosella* plants were cleaned and selected manually, the separated stems were cut into small sizes of approximately 0.5 cm and dried at 60 °C to constant weight, and then subjected to grinding in a planetary ball mill PM 100, Retsch, Haan, Germany, and sieving in an AS 200 analytical sieve shaker, Retsch, Haan, Germany. The materials of the 250 and 500 µm fractions (T1) were retained, to which chemical treatments were applied with 200 mL of sulfuric acid (H_2_SO_4_) at 1.25% *w*/*v* (T2), alkaline treatments with 200 mL of sodium hydroxide (NaOH) at 2% *w*/*v* (T3) and sequential mixed treatments (T4) of 200 mL of H_2_SO_4_ and 200 mL of NaOH in the concentrations described above, for 60 min and 120 °C under constant agitation. Subsequently, the pre-treated biomasses were vacuum filtered, and washed with distilled water until neutral pH was achieved in order to eliminate other soluble substances. Finally, the biosorbents obtained were dried at 50 °C for 16 h and kept in a desiccator until use. The plant material and biosorbents obtained are shown in Figure 2.

### 2.4. Point of Zero Charge

It was determined by adjusting the pH of the solutions to 3, 4, 5, 6, 7, 8, 9 and 10 with 0.1 N NaOH and 0.1 N HCl, then adding 0.5 g to each adjusted solution of the untreated biomass fraction of 250 and 500 µm, for 24 h at 150 rpm and 25 °C under constant agitation. After the time, the samples were filtered and the pH was measured to obtain the point of zero charge (PCZ) [33].

### 2.5. Total Organic Carbon

The samples of the modified biosorbents were previously homogenized, then 50 mg of each material was weighed in ceramic containers, to be later analyzed in a total organic carbon analyzer TOC-L CSN-SSM 5000A, Shimadzu, Kyoto, Japan, with a flow of oxygen of 150 mL/min through the software TOC control L V. 1.07.

### 2.6. Heavy Metal Biosorption

A homogeneous stock solution of heavy metals (As^3+^, Cd^2+^, Pb^2+^ and Zn^2+^) was prepared in ultrapure water to obtain concentrations of 10 mg/L of each metal in the solution mix; all reagents were obtained from Sigma-Aldrich (Taufkirchen, Germany), and the solution was stored in an amber glass bottle in the absence of light, until before its use in the experiments.

Heavy metal biosorption tests were done discontinuously at a controlled temperature of 20 °C, and all the glassware used was treated with a 0.1 N HNO_3_ solution before the experiments. The pH of the solution was adjusted to 5.0 with 0.1 N HNO_3_ and 0.1 N NaOH, using a Lab 885 calibrated potentiometer, SI Analytics, Germany. A known amount of biosorbent (0.2 g dissolved in 800 mL) was worked on in 1 L beakers with constant stirring at 150 rpm for 120 min (samples were extracted every 30 min). Subsequently, the biosorbent was separated from the solution by filtration (0.45 µm), and the reading was carried out in an inductively coupled plasma optical emission spectrophotometer (ICP-OES), 9820, Shimadzu, Kyoto, Japan, and the analysis was carried out in axial mode, with a sample exposure of 30 s and an argon gas flow of 10 L/min. The removal rates of heavy metals were computed using the following equations:

(1) $$R_{e}\%=\frac{\left(c_{o}-c_{t}\right)}{c_{o}}$$

(2) $$q_{t}=\frac{\left(c_{o}-c_{t}\right)\cdot v}{m}$$

where *R_e_*% is the percentage of metal ion removal, $q_{t}$ is the amount of adsorbed metal cations, $c_{o}$ (mg L^−1^) is the initial concentration of the metal ion, $c_{t}$ (mg L^−1^) is the equilibrium concentration of the metal ion, v (L) is the volume of the aqueous solution and m (g) is the amount of dry biosorbent.

### 2.7. pH Influence

A total of 0.2 g of the 250 and 500 µm fractions of the T4 biosorbent was taken because of its high percentages in removing heavy metals (As^3+^, Cd^2+^, Pb^2+^ and Zn^2+^). After that, the weighed biomasses were mixed with 800 mL of a mixed solution with a concentration of 10 mg/L for each metal. Then, the pH of the solutions was adjusted to 3, 4, 5 and 6 with 0.1 N HNO_3_ and 0.1 N NaOH, using a calibrated potentiometer Lab 885, SI Analytics, Mainz, Germany. The tests were carried out at a controlled temperature of 20 °C and kept under agitation at 150 rpm for 120 min. Finally, the solutions were filtered using 0.45 µm nylon membrane filters; and the resulting solutions were measured using an inductively coupled plasma optical emission spectrophotometer, ICP-OES 9820, Shimadzu, Kyoto, Japan; the analysis was performed in axial mode, with a sample exposure of 30 s and an argon gas flow of 10 L/min.

### 2.8. Adsorption Isotherms

To prepare the solutions, 0.05 g of T4 biosorbent (250 and 500 µm) was added to 200 mL of a multimetal solution (As, Cd, Pb and Zn) with concentrations of 1, 5, 10, 25, 50, 100, 125 and 150 mg/L; the pH of the solution was adjusted to 5.0 with 0.1 N NaOH and 0.1 N HNO_3_; and the solutions were stirred at 150 rpm for 120 min at 20 °C. Then, the mixtures were filtered at 0.45 µm, and heavy metal concentrations were determined by ICP-OES 9820, Shimadzu, Kyoto, Japan, calibrated with As, Pb, Cd and Zn standards (Calibration solution traceable, Sigma-Aldrich, Taufkirchen, Germany). The analysis was performed in axial mode, with a sample exposure of 30 s and an argon gas flow of 10 L/min.

Two models have been tested: Langmuir and Freundlich in order to evaluate the biosorption efficiency of heavy metals studied.

(3) $$\mathrm{Langmuir}:\;qe=\frac{\left(q_{m}\cdot K_{L}\cdot Ce\right)}{\left(1+K_{L}\cdot Ce\right)}$$

(4) $$\mathrm{Freundlich}:\;qe=K_{F}\cdot Ce^{1/n}$$

where qe (mg g^−1^) is the amount of metal adsorbed at equilibrium, $q_{m}$ (mg g^−1^) is the maximum heavy metal biosorption capacity of the biosorbent, $K_{L}$ is the adsorption equilibrium constant of Langmuir, Ce (mg L^−1^) is the adsorbate equilibrium concentration and $K_{F\;}$ and 1/n are constants of Freundlich model.

### 2.9. Adsorption Kinetics

To prepare the solutions, 0.05 g of T4 biosorbent (250 and 500 µm) was added to 200 mL of a multimetal solution (As, Cd, Pb and Zn) with a concentration of 25 mg/L. The pH of the solution was adjusted to 5.0 with 0.1 N NaOH and 0.1 N HNO_3_, and the solutions were stirred at 150 rpm for 120 min at 20 °C. The solutions were withdrawn at 0, 5, 10, 20, 30, 45, 60, 90, 120, 180, 240 and 300 min. Then, the mixtures were filtered at 0.45 µm, and heavy metal concentrations were determined by ICP-OES 9820, Shimadzu, Kyoto, Japan, calibrated with As, Pb, Cd and Zn standards (Calibration solution traceable, Sigma-Aldrich, Taufkirchen, Germany). The analysis was performed in axial mode, with a sample exposure of 30 s and an argon gas flow of 10 L/min.

Pseudo-first order and pseudo-second order mathematical models were used to evaluate the adsorption kinetics.

(5) $$\mathrm{Pseudo}\text{-}\mathrm{first}\;\mathrm{order}:\;qt=qe\cdot \left(1-e^{k_{1}*t}\right)$$

(6) $$\mathrm{Pseudo}\text{-}\mathrm{second}\;\mathrm{order}:\;qt=\frac{\left(k_{2}\cdot qe^{2}\cdot t\right)}{\left(1+k_{2}\cdot qe\cdot t\right)}$$

where qt (mg g^−1^) is the amount of metal adsorbed at time t (min), qe (mg g^−1^) is the amount of metal adsorbed at equilibrium, and *k*_1_ and *k*_2_ are the constants of the pseudo-first and pseudo-second order models.

### 2.10. FTIR Analysis

The initial and final functional groups of the 250 and 500 µm fractions were recorded in a Fourier transform infrared spectrophotometer (FTIR), Nicolet IS50, ThermoFisher, Waltham, MA, USA, using 0.1% KBr pressed tablets; readings were made with the modulus of transmission in the wavenumber range of 400 to 4000 cm^−1^, with a resolution of 8 cm^−1^ and 32 scans.

### 2.11. Thermal Analysis

A total of 10 mg of sample of the biosorbents of 250 and 500 µm was weighed to determine the thermal stability of the materials by TGA (thermogravimetric analysis) and DTA (differential thermal analysis), in a temperature range between 20 and 600 °C, in a N_2_ atmosphere, with a speed of heating at 10 °C/min, using a TGA 550 thermal analyzer, TA Instrument, New Castle, DE, USA.

### 2.12. Morphological Analysis

The geometry of the biosorbents was analyzed using a stereomicroscope SMZ-161T, Motic, Schertz, TX, USA. The measurements of length and diameter of the polymeric materials were calculated with the Motic Imagen Plus software of the equipment above.

### 2.13. Statistical Analysis

All data were obtained in triplicate, and an analysis of variance (ANOVA) and Tukey’s multiple range test at 95% confidence were used. Origin Pro 2022 software (OriginLab Corporation, Northampton, MA, USA) was used.

## 3. Results and Discussion

### 3.1. Characterizations of Bioadsorbents

#### 3.1.1. Proximate Analysis of Vegetal Material

The water content in the fresh stem was 82.12%, which allowed one to have an approximation of the porosity of the material; in the case of the dry samples, the humidity of the stem was 7.80%, which was higher compared to the leaf and seeds of *R. acetosella* (Table 1). On the other hand, of particular interest were the fiber contents, which presented a significant difference (*p* < 0.05), which in the case of the dry stem had a higher value (62.28%). That is why it was decided to obtain the biosorbents from this fraction of the plant, rich in cellulose, hemicellulose, and lignin, which are essential in the adsorption of heavy metals [34,35,36,37,38].

#### 3.1.2. Point of Zero Charge

**Figure 3:**
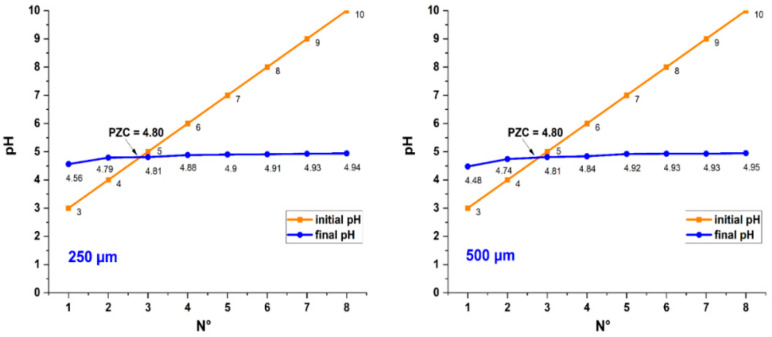
Point of zero charge in the fractions of 250 and 500 µm.

The determination of the PCZ was carried out to know the pH value from which the metallic cations (As, Cd, Pb and Zn) could be adsorbed. In Figure 3, it can be verified that pH 4.8 was the neutral charge point of the untreated biomass for both fractions; in the case of solutions with values higher than PCZ, the adsorption of cations would be favored due to the presence of negative charges [39,40,41,42,43,44]. Natural fibers have acid groups on their surface; they completely dissociate above pH 4.0, which would favor the adsorption of different molecules. It is also known that cellulose fibers have the presence of hydroxyl groups, which allows for the formation of hydrogen bonds with other compounds; to achieve those above, different chemical treatments for the biomass must be developed [45,46]. However, modification of the pH would improve the adsorption capacity of the biosorbents under study, although, at a more alkaline pH, metals have the tendency to precipitate as metal hydroxide [47,48].

#### 3.1.3. Total Organic Carbon

Significant amounts of total organic carbon were found in all biosorbents. In Table 2, values between 22.86 and 25.66% are reported for the 250 µm retained fraction and between 22.59 and 24.08% for the 500 µm retained fraction. Significant differences were observed in most cases (*p* > 0.05). The presence of adequate amounts of carbon is essential in heavy metal biosorption processes [49], since they are associated with the presence of lignocellulosic polymeric compounds such as cellulose, hemicellulose and lignin [34].

### 3.2. Application of Bioadsorbents

#### 3.2.1. Heavy Metal Biosorption

Batch experiments were performed to determine the removal capacity of the heavy metals studied, using modified biomass of *R. acetosella*. Table 3 shows the removal percentages for As, Cd, Pb and Zn, in a multi-metal system and initial concentrations of 10 mg/L for each ion. It was observed that the biosorbents presented a good affinity for the metal cations, for a contact time of 120 min, especially the 250 µm fraction that allowed one to remove between 11.80 and 32.50% of As, 11.17 and 34.10% of Cd, and 12.23 and 36.30% of Zn, Pb being the one that presented the greatest removal between 90.70 and 96.14%, similar to values reported by various authors, using different lignocellulosic materials [50,51,52,53]. On the other hand, the mixed treatment presented better heavy metal adsorption capacity, which would be related to the acid treatment that eliminated hemicellulose and the basic treatment that removed lignin, obtaining biosorbents rich in cellulose, a fundamental component in the processes of metal cation removal [54,55].

**Figure 4:**
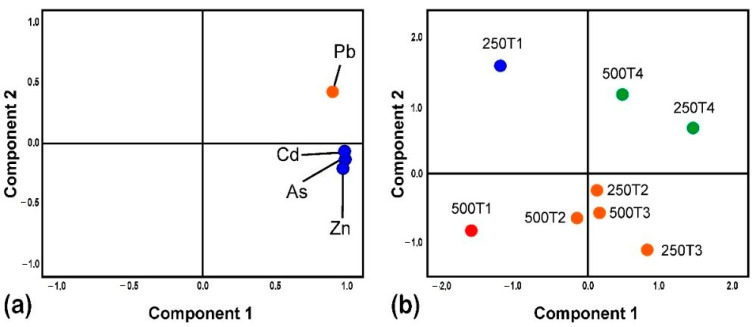
Principal component analysis in the removal of heavy metals, metal cations (**a**) and biosorbents (**b**).

Figure 4 shows the principal component analysis (PCA) for heavy metals and biosorbents, confirming the differences shown in Table 3.

As far as metallic cations were concerned (Figure 4a), it is possible to distinguish between a first group made up of Cd, As and Zn (associated with component 1), and a second group composed of Pb (associated with component 2). In regard to polymeric biosorbents (Figure 4b), two different groups were observed, the first one is composed of mixed treatments (250T4 and 500T4), and the other was made up of acid and basic treatments (250T2, 250T3, 500T2 and 500T3) and independently biosorbents *in nature* 250T1 and 500T1. The PCA is a statistical technique for reducing factors and allows one to appreciate the relationship of complex variables [21].

**Figure 5:**
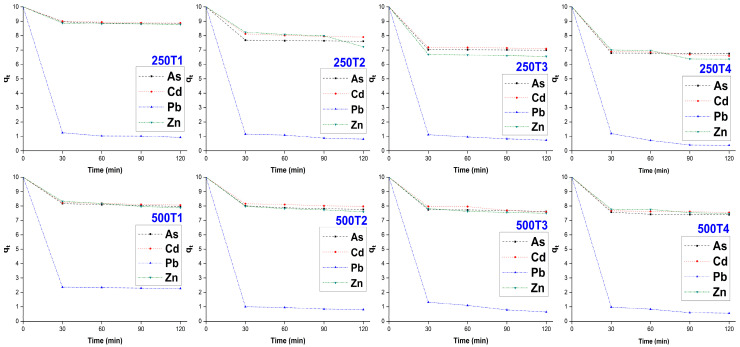
Variation of heavy metal biosorption over time in the fractions of 250 and 500 µm.

The treatments T1, T2, T3 and T4 in the fractions of 250 and 500 µm showed a significant difference in most cases (*p* < 0.05). It was also observed that the adsorption of heavy metals decreased over time, noting that the highest rate of decrease in the initial concentration occurred in the first 30 min; this behavior was more significant in the case of Pb. Nevertheless, for longer contact times, a decrease in the removal of all metals was evidenced, as shown in Figure 5. Other authors reported similar results for equilibrium times between adsorbent and adsorbate, which varied between 5 and 40 min, using solutions contaminated with different heavy metals and biosorbents of crambe [41], cashew-nut shell [56], cassava peel [57] and pinus bark [58]; likewise, it was observed that the adsorption kinetics is influenced by chemical modifications to the biomass [59,60], so the removal rates of biosorbents T2, T3 and T4 were higher than biosorbent T1.

#### 3.2.2. pH Influence

**Figure 6:**
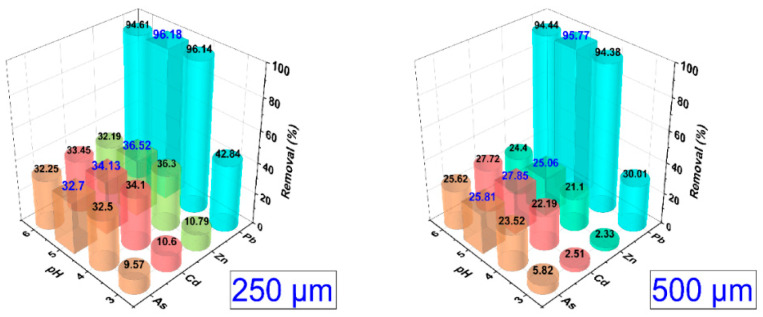
Influence of pH on the removal of heavy metals for the 250 and 500 µm fractions of the mixed treatment T4.

Figure 6 shows that pH 5 had the best heavy metal removal percentages, with the 250 µm fraction of the mixed treatment T4 showing the best performance, with removal rates of 32% of As, 34.13% of Cd, 36.52% of Zn, and 96.18% of Pb. In the case of the 500 µm fraction, lower values of 25.81% As, 27.85% Cd, 25.06% Zn, and 95.77% Pb were obtained, observing that above pH 5, a decrease in the removal of the metal cations studied was reported for both fractions.

The better removal of the 250 µm fraction was due to the greater contact surface that the biosorbents had during the sorption process, additionally enhanced by the fact that they were treated sequentially with sulfuric acid and sodium hydroxide, which caused the removal of hemicellulose and lignin, respectively [54,55]. Similar results were reported by several authors for pretreated biomasses rich in lignocellulosic components [41,42,61].

#### 3.2.3. Adsorption Isotherms

It was developed to establish, in equilibrium, the relationship between the metal cations and the biosorbent T4 (250 and 500 µm), and the parameters for the optimal use of the material and the design of possible biosorption systems were established [61,62]. Table 4 shows the results where it could be seen that the Langmuir isotherms showed the best R^2^ values (between 0.955 and 0.98), which suggests the occurrence of heavy metal adsorption at the monolayer level [57].

In the case of Pb, high removal rates were found, especially for the T4 biosorbent (250 µm fraction). The qm value was 156.25 mg/g, lower than rose stems (344.82 mg/g) reported for a mixed biosorbent, treated sequentially with acid and base [54]. On the contrary, the result found in the present study was higher than that of a biosorbent obtained from *Lavandula pubescens* Decne, whose qm value was 91.12 mg/g [63].

**Figure 7:**
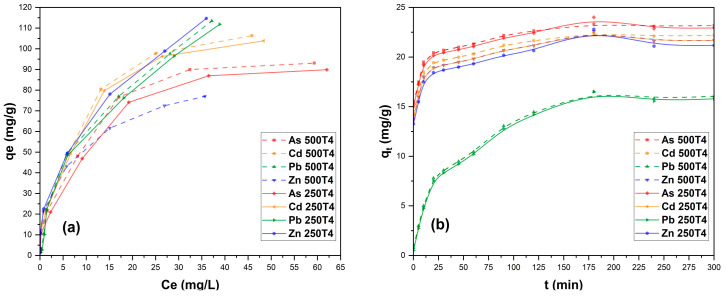
Langmuir adsorption isotherms (**a**) and pseudo-second order model (**b**), for T4 (250 and 500 µm).

As far as the removal of Zn, Cd, and, As was concerned, values of qm between 95.24 and 117.65 mg/g (250 and 500 µm) were found, which were lower than Pb (156.25 mg/g). However, they were higher than other biosorbents such as peanut shell adsorbents [64], cassava peel [57], *Acacia nilotica* stem [65], *Colocasia esculenta* stem [66], and maize stalks [67]. Figure 7 shows the mathematical modeling of the Langmuir isotherm and the pseudo-second order.

#### 3.2.4. Adsorption Kinetics

Table 5 shows the results of the kinetic parameters obtained from the linearization of the pseudo-first order and pseudo-second order models. The results of all the kinetic models evaluated show that the pseudo-second order model presented the best R^2^ values (between 0.986 and 0.999) for the adsorption of As, Cd, Pb, and Zn; similar values were reported for cassava peel biosorbents [57], *Platanus orientalis* Linn leaves [68], guava leaf [69], and rose stems [54]. The data indicate that there is chemical adsorption by the formation of chemical bonds, between the biosorbents and metal cations at the monolayer level [70,71,72], since there would be electron exchange between the sorbent and the adsorbate, through covalent and ion exchange bonds, which would allow for the initial formation of a single layer by the action of the active centers of the biosorbent, with the possibility of the subsequent formation of other layers by physisorption [57,71].

### 3.3. Characterization before and after Adsorption

#### 3.3.1. FTIR Analysis

**Figure 8:**
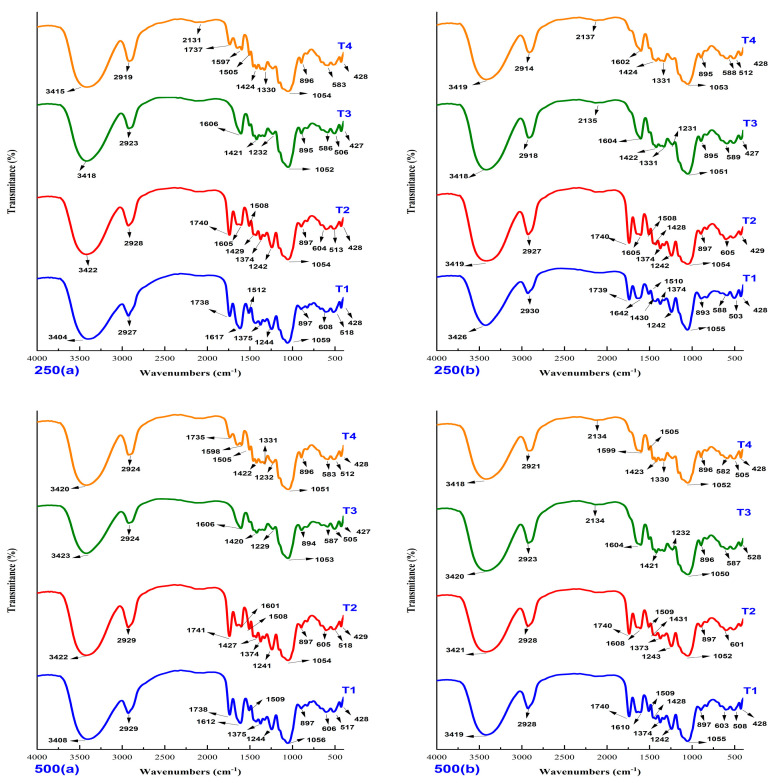
FTIR infrared spectra of the 250 and 500 µm fractions, before (**a**) and after (**b**) the biosorption process.

Figure 8 shows the vibrational stretching before and after the heavy metal biosorption process, in the ranges of 3408–3426, 2914–2930, 2131–2137, 1602–1741, 1420–1599, 1229–1375, 1050–1059 and 427–897 cm^−1^. FTIR analysis suggests the existence of different functional groups in the modified cellulosic biosorbents [73,74,75]. Many of the peaks before the process were also observed at the end of the biosorption, also noting that the untreated material presents many of the functional groups seen in the chemically treated biosorbents; these peaks could have contributed to the adsorption of the heavy metals As, Cd, Pb and Zn.

More specifically, the peaks between 3408 and 3426 cm^−1^ would correspond to the -OH hydroxyl groups of celluloses, proteins, and pectic compounds, and the peaks from 2914 to 2930 cm^−1^ would represent the tension band of the -C-H bond, the peaks between 1229 and 1741 cm^−1^ would indicate the presence of carboxylic groups -COOH or derivatives, such as carboxylates, the peaks of 1050–1059 cm^−1^ would correspond to the C-O bond frequently of the ether group -C-OC- and of the C-OH found in celluloses, the peaks from 893 to 896 cm^−1^ would represent a weak tension band also assigned to carboxyl groups and the peak from 427 to 897 cm^−1^ would represent the presence of alkene groups C=C-H or Ar-H, the latter of which is, above all, abundant in cellulose chains.

The aforementioned was observed with greater emphasis in the mixed treatment, which is the one that obtained the highest removal percentages. It is important to mention that the band changes after the pretreatments showed that different functional groups could have contributed to the biosorption process. Furthermore, new sorption bands were recorded after interaction with H_2_SO_4_ and NaOH.

After the interaction of the mixed biosorbent T4 with the metal cations, a greater modification of the spectrum was observed with respect to the original, due to the high removal of heavy metals, in which there is a certain shift to the right in the peaks corresponding to the band tension of the carboxyl groups (-C=O and -C=OH), which would confirm the preferential participation of these functional groups in the union with metals. In fact, different authors have frequently highlighted the involvement of these groups in metal uptake by various biomasses [39,76,77,78], which could also be attributed to the tension band of the -C-H bond [63,79,80].

The peaks at 1602–1741 cm^−1^ could be attributed to the amide group [81,82]. The bands observed in the modified biosorbents would then be attributed mainly to the chemical groups OH, NH, -C-H, COOH and C-O [83,84,85]. On the other hand, the OH and COOH functional groups are probably the most important participating groups in the biosorption process. In particular, the COOH group would be an indicator of the interaction and chelating reaction with metallic cations such as As, Cd, Pb and Zn [83]. Furthermore, it is known that hydroxyl and carboxyl groups are present in cellulose and hemicellulose polymers, and they are structural components of the cell wall of plants. Thus, adsorption on inert biomasses is due to weak electrostatic interactions between adsorbate and adsorbent [23].

#### 3.3.2. Thermal Analysis

**Figure 9:**
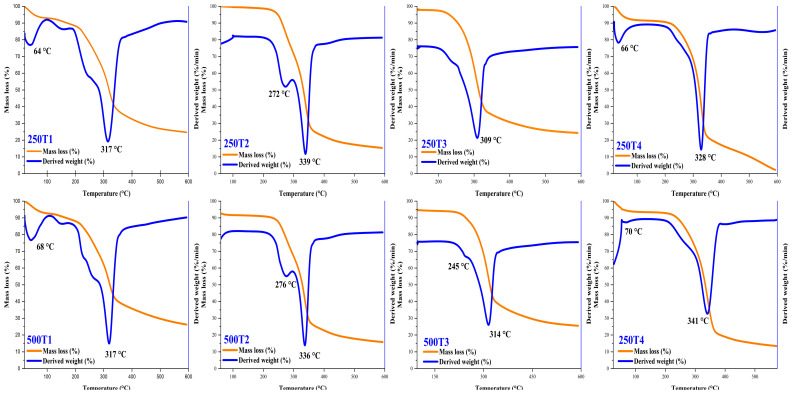
TG and DTG curves of the biosorbents obtained from the fractions of 250 and 500 µm.

The *R. Acetosella* biosorbents had similar thermal behaviors (Figure 9), and similar TGA and DTG curves were observed for the 250 and 500 µm fractions. Similar behaviors were also appreciated with other materials rich in cellulose, hemicellulose, and lignin, both in events and temperature ranges reported in the literature.

T1 exhibited two different events, first at 64 and 68 °C, with a mass loss of around 5% of the material, mainly due to the loss of water and other volatile compounds. The second event occurred at 317 °C, with a mass loss of about 46%, due to the decomposition of organic compounds such as lignin, cellulose, and hemicellulose of the particulate material [86]. 

In the case of T2, the decomposition of organic components such as lignin, cellulose, and hemicellulose is observed at 272 and 276 °C [87], with a loss of 15% of the biosorbent mass. The second occurrence occurred at 336 and 339 °C, which would be associated with the final degradation of the lignocellulosic matrices and ash residues of the non-incinerated components, which gave rise to a 57% loss in material mass [41].

For the modified biosorbent T3, it was observed that between 309 and 314 °C the incineration of compounds such as cellulose, hemicellulose, and lignin occurs, with a mass loss of 52%; above these temperatures, non-volatile inorganic material is observed [88].

As far as treatment T4 was concerned, two different events were observed: first, at 66 and 70 °C water and other volatile substances were eliminated, with a mass loss of 4 and 5%. The second event occurred between 328 and 341 °C, where most of the cellulosic compounds are lost, which causes a weight loss of between 59 and 60%. At higher temperatures, the rest of the cellulosic matrix is lost [89].

#### 3.3.3. Morphological Analysis

**Figure 10:**
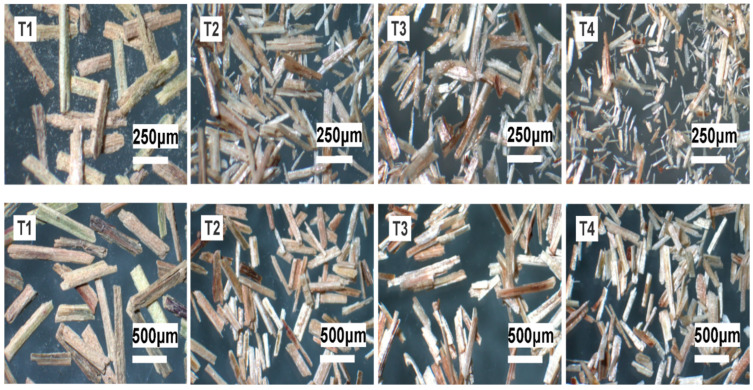
Geometry of the obtained biosorbents (white bars indicate 250 and 500 µm).

In Figure 10, it is observed that the geometry of all the biosorbents presented the shape of rods, with different lengths and diameters. It can also be seen that the chemical treatments caused a reduction in the initial dimensions, as well as a cracking on the surface of the treated materials [49,61], which possibly contributed to the improvement of the adsorption capacity of the heavy metals studied.

The average values of the measurements and the aspect ratio between length and diameter can be seen in Table 6, in which it can be seen that the chemical treatments carried out allowed the measurements of the biosorbents to be reduced [86], obtaining a larger surface area contact by the elimination of compounds such as hemicellulose and lignin [54,90]. In the case of the mixed T4 treatment, a greater loss of the original measurements was observed, due to the sequential combination of sulfuric acid and sodium hydroxide [91,92,93]. The surface of the biosorbent before the chemical modification was rough and irregular; after the treatment, a greater cracking was observed on the surface, which allowed it to be more porous, thus allowing a more available surface to capture the metal ions As, Cd, Pb and Zn [81,94,95]. Lately, the use of metallic nanoparticles in wastewater treatment has been investigated because their properties are closely linked to their size and morphology, making them highly reactive due to their large surface area [24].

## 4. Conclusions

This article highlights the potential use of the weed *Rumex acetosella*, in obtaining biosorbents rich in fiber and total organic carbon chemically modified, which allowed us to improve the removal capacity of heavy metals in polluted waters, due to the presence of various functional groups, especially OH, NH, -C-H, COOH, and C-O, which contributed to the selective removal of up to 96. The results were as follows: 96.14% of Pb, 36.30% of Zn, 34.10% of Cd, and 32.50% of As, with the fraction of 250 µm and mixed treatment, for contact times from 30 to 120 min and optimum pH of 5.0.

The thermal behavior of the materials obtained, between 20 and 600 °C, was similar to that of other cellulosic biosorbents. It was also observed that, at the beginning, the surface of the in natura material was rough and irregular, and that mainly after the mixed treatment, greater cracking of the microstructure was observed, which allowed for obtaining a topography with greater chelating capacity. The Langmuir and pseudo-second order models were better fitted to the adsorption data. 

The use of new waste raw materials, as in the case of the plant material studied, allows for obtaining biosorbents with high removal rates of metal cations. In particular, the sequential acid-base treatment was highlighted. On the other hand, the biosorbents obtained could be used in the treatment of various wastewaters, in a simple, profitable, and eco-friendly way.

## Figures and Tables

**Table 1:** Proximal analysis of *R. acetosella* (%).

Part of Plant	Moisture		Protein		Fat		Ash		Fiber		Carbohydrates	
	$\bar{x}\;\pm \;s$	*	$\bar{x}\;\pm \;s$	*	$\bar{x}\;\pm \;s$	*	$\bar{x}\;\pm \;s$	*	$\bar{x}\;\pm \;s$	*	$\bar{x}\;\pm \;s$	*
Stem	7.80 ± 0.51	a	7.24 ± 0.73	a	1.59 ± 0.22	a	3.28 ± 0.32	a	62.28 ± 0.11	a	80.09 ± 0.64	a
Leaf	7.52 ± 0.43	a	9.85 ± 0.32	b	2.16 ± 0.41	a	5.25 ± 0.35	b	58.33 ± 0.61	b	80.47 ± 0.43	a
Seeds	7.39 ± 0.46	a	10.45 ± 0.12	b	3.90 ± 0.77	b	7.41 ± 0.54	c	30.85 ± 0.31	c	70.85 ± 0.63	b

* Evaluated by Tukey test at 95% confidence, different letters indicate significant difference.

**Table 2:** Total organic carbon (%).

Treatments	Biosorbent (250 µm)		Biosorbent (500 µm)	
	$\bar{x}\;\pm \;s$	*	$\bar{x}\;\pm \;s$	*
T1	25.66 ± 1.23	a	24.08 ± 1.54	a
T2	23.39 ± 0.11	b	23.49 ± 1.00	a
T3	25.04 ± 0.20	a	23.83 ± 0.83	a
T4	22.86 ± 0.38	b	22.59 ± 0.65	a

* Evaluated by Tukey test at 95% confidence, different letters indicate significant difference.

**Table 3:** Heavy metal removal (%).

Biosorbent.	Treatments	As		Cd		Pb		Zn	
		$\bar{x}\;\pm \;s$	*	$\bar{x}\;\pm \;s$	*	$\bar{x}\;\pm \;s$	*	$\bar{x}\;\pm \;s$	*
250 µm	T1	11.80 ± 1.25	a	11.17 ± 1.44	a	90.70 ± 1.22	a	12.23 ± 1.03	a
	T2	23.87 ± 2.03	b	21.03 ± 3.03	b	91.89 ± 0.84	a	27.73 ± 2.04	b
	T3	30.10 ± 1.33	c	29.10 ± 1.63	c	92.65 ± 1.02	a	34.47 ± 1.01	c
	T4	32.50 ± 2.14	c	34.10 ± 1.15	d	96.14 ± 1.12	b	36.30 ± 0.05	c
500 µm	T1	10.73 ± 0.16	a	10.93 ± 0.65	a	86.90 ± 0.42	a	11.70 ± 0.37	a
	T2	23.30 ± 0.16	b	20.20 ± 0.57	b	90.91 ± 0.26	b	25.80 ± 0.27	b
	T3	23.90 ± 0.65	b	23.97 ± 1.24	c	91.45 ± 0.31	b	26.63 ± 0.37	c
	T4	25.97 ± 0.47	c	24.47 ± 0.75	c	94.38 ± 0.39	c	27.30 ± 0.54	c

* Evaluated by Tukey test at 95% confidence, different letters indicate significant difference.

**Table 4:** Parameters of the adsorption isotherms for the T4 biosorbent (250 and 500 µm fractions).

Biosorbent	Heavy Metal	Langmuir Isotherm			Freundlich Isotherm			
		*q_m_* (mg/g)	*K_L_* (L/mg)	*R* ^2^	*K_F_*	1/*n*	*n*	*R* ^2^
250T4	As	95.24	0.21	0.976	13.25	0.575	1.74	0.951
	Cd	109.89	0.25	0.975	16.93	0.581	1.71	0.930
	Pb	156.25	0.06	0.981	8.04	0.840	1.19	0.946
	Zn	119.05	0.23	0.955	17.96	0.591	1.69	0.907
500T4	As	97.09	0.27	0.979	15.46	0.557	1.79	0.934
	Cd	112.36	0.26	0.974	17.78	0.580	1.72	0.936
	Pb	140.85	0.09	0.964	9.92	0.783	1.19	0.956
	Zn	117.65	0.26	0.957	19.06	0.575	1.74	0.915

where: 250T4, mixed treatment in the 250 µm fraction; 500T4, mixed treatment in the 500 µm fraction.

**Table 5:** Parameters of the adsorption kinetic models for the biosorbent T4 (250 and 500 µm fractions).

Biosorbent	Heavy Metal	Pseudo First Order			Pseudo Second Order		
		*q_e_* (mg/g)	*K* (min^−1^)	*R* ^2^	*q_e_* (mg/g)	*K_2_* (g mg^−1^ min^−1^)	*R* ^2^
250T4	As	6.17	0.0122	0.873	23.47	0.282	0.998
	Cd	5.96	0.0133	0.886	22.12	0.273	0.998
	Pb	14.11	0.0149	0.966	17.24	0.038	0.986
	Zn	6.68	0.0105	0.849	21.93	0.242	0.996
500T4	As	5.47	0.0179	0.929	23.36	0.325	0.999
	Cd	5.52	0.0172	0.923	22.42	0.305	0.999
	Pb	13.97	0.0158	0.985	17.33	0.040	0.988
	Zn	6.04	0.0128	0.880	22.17	0.270	0.998

where: 250T4, mixed treatment in the 250 µm fraction; 500T4, mixed treatment in the 500 µm fraction.

**Table 6:** Measurements of the modified biosorbents obtained.

Treatments	Biosorbents of the Fraction of 250 µm			Biosorbents of the Fraction of 500 µm		
	Length (µm) $\bar{x}\;$± s	Diameter (µm) $\bar{x}\;\pm \;s$	L/D $\bar{x}\;\pm \;s$	Length (µm) $\bar{x}\;\pm \;s$	Diameter (µm) $\bar{x}\;\pm \;s$	L/D $\bar{x}\;\pm \;s$
T1	340.03 ± 58.65	69.11 ± 8.02	4.99 ± 1.04	750.01 ± 33.42	152.74 ± 7.26	4.92 ± 0.34
T2	256.38 ± 57.21	38.21 ± 8.17	6.79 ± 1.25	464.48 ± 61.65	84.18 ± 19.99	5.76 ± 1.29
T3	267.11 ± 61.20	54.90 ± 10.18	4.96 ± 1.16	685.67 ± 81.29	105.23 ± 18.97	6.63 ± 1.30
T4	194.83 ± 38.20	31.72 ± 8.42	6.38 ± 1.47	405.64 ± 57.36	74.82 ± 9.18	5.50 ± 1.18

where: L/D, aspect ratio of the biosorbents obtained.

## Data Availability

The data presented in this study are available in this same article.
